# Widespread pustular rash in an elderly patient

**DOI:** 10.1016/j.jdcr.2024.09.008

**Published:** 2024-09-29

**Authors:** Cristina Grechin, Emily Orr, Síona Ní Raghallaigh, Marina O’Kane

**Affiliations:** Dermatology Department, Beaumont Hospital, Dublin, Ireland

**Keywords:** biologic therapy, GPP, therapeutic and clinical challenge

An 89 year-old woman was hospitalized with acute onset of fever and generalized confluent erythematous plaques and pustules (Fig 1), preceded by administration of terbinafine (24 days prior) and furosemide (7 days prior). There was no personal or family history of psoriasis. However, we subsequently obtained a history suggestive of pustular acrodermatitis treated by her primary care physician as a fungal infection.

**Fig 1:**
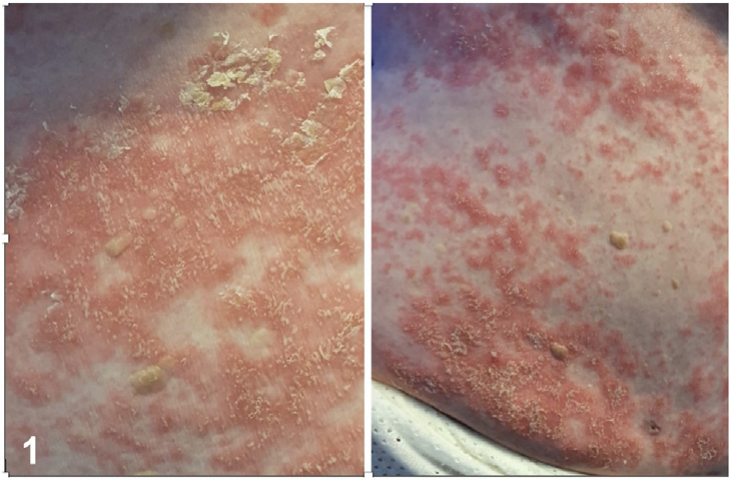

Skin biopsy revealed acanthotic epidermis with moderate spongiosis and exocytosis of neutrophils, subcorneal pustules, and upper dermal interstitial and perivascular inflammation with dermal edema. Viral and bacterial swabs were negative. Despite discontinuation of terbinafine and furosemide, followed by treatment with oral prednisolone 40 mg daily and acitretin 20 mg daily over the next 3 months, her rash persisted (Fig 2).

**Fig 2:**
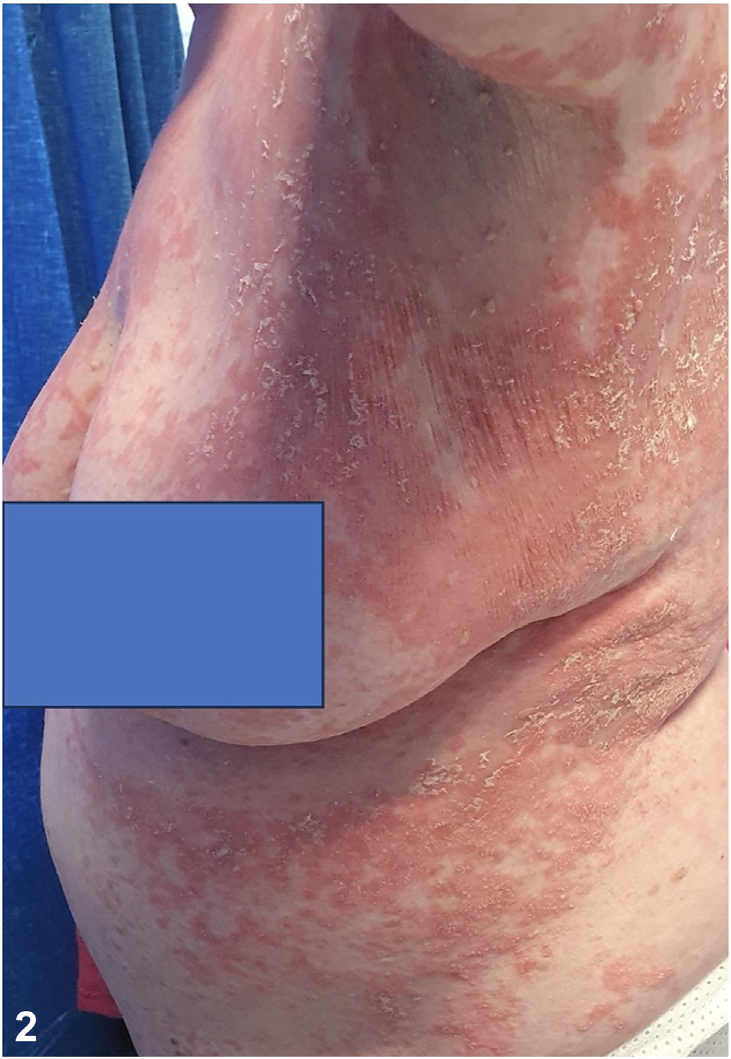

**Question 1: What is your diagnosis?**

A. Acute generalized exanthematous pustulosis (AGEP)
B. Generalized pustular psoriasis (GPP)
C. IgA pemphigus
D. Subcorneal pustular dermatosis (SPD)
E. Bullous tinea

**Answers:**

A. Acute generalized exanthematous pustulosis (AGEP) – Incorrect. AGEP is a severe cutaneous adverse reaction. More than 90% of cases are attributable to initiation of a drug. AGEP typically resolves spontaneously (within 2 weeks) without recurrence if the culprit drug is stopped.^1^^,^^2^ However, AGEP shows significant clinical and histopathologic overlapping features with GPP, causing diagnosis and treatment challenges to clinicians.^1^
B. Generalized pustular psoriasis (GPP) – Correct. GPP is a systemic inflammatory disease characterized by cutaneous erythema and macroscopically visible sterile pustules on an erythematous base, not restricted to acral regions or psoriatic plaques.^3^ GPP is classically associated with a protracted, relapsing remitting course.^1^^,^^3^ GPP pathogenesis primarily involves abnormal activation of the interleukin (IL)-36 pathway of the innate immune system with secondary contributions from the adaptive immune system.^2^
C. IgA pemphigus – Incorrect. IgA pemphigus is a rare autoimmune disease that typically presents with flaccid pustules on an erythematous base that ruptures to form annular crusts over the plaque. It has a subacute onset, with flexural areas being involved.^2^
D. Subcorneal pustular dermatosis (SPD) – Incorrect. SPD is an asymptomatic eruption of grouped pustules that evolves into half-pustular, half-clear fluid blisters and coalesces to form annular or serpiginous patterns. Typically has more subacute onset and evolution than AGEP and GPP and responds to dapsone.^2^
E. Bullous tinea – Incorrect. Bullous tinea is a rare dermatophyte infection that typically presents with inflammatory subcorneal or intraepidermal vesicles and bullae on the soles.^1^ Clinically, it can appear similar to a localized patch of AGEP or PP given the pustules on an erythematous base.^1^

**Question 2: What is the clinical course of GPP?**

A. GPP has a relapsing remitting course or persisting with intermittent flares
B. Mortality rate in GPP is >50%
C. Older patients have a better prognosis compared with younger
D. In most cases, GPP is not associated with any systemic complications
E. Most patients will not have any recurrence of the disease

**Answers:**

A. GPP has a relapsing remitting course or persisting with intermittent flares – Correct. The clinical course of GPP is heterogeneous with a relapsing remitting course or persisting with intermittent flares. The course and symptom severity may vary with each flare.^1^^,^^3^
B. Mortality rate in GPP is >50% – Incorrect. The literature highlights that the mortality rate ranges from 4% to 24%.^3^ The most common causes of mortality include sepsis and multiorgan failure.^2^
C. Older patients have a better prognosis compared with younger – Incorrect. Particularly in older patients, GPP can be life-threatening and about 5% to 10% of GPP flares are fatal.^4^
D. In most cases, GPP is not associated with any systemic complications – Incorrect. GPP flares are frequently accompanied by systemic symptoms, including fever, malaise, and leukocytosis and may lead to hospitalization and life-threatening complications.^1^^,^^5^ The complications and morbidity from GPP include pneumonia, liver damage, hypoalbuminemia, hypocalcaemia, renal tubular necrosis, malnutrition, and cardiorespiratory failure.^3^^,^^4^
E. Most patients will not have any recurrence of the disease – Incorrect. GPP is a potentially life-threatening disease that it may present with repeated acute flares involving systemic inflammation or as a chronic disease with intermittent flares.^3^^,^^5^

**Question 3: Regarding GPP treatment, which of the following is correct?**

A. There are globally approved guidelines for the treatment of GPP
B. Spesolimab, an anti-IL-36 receptor antibody, is the most specific agent and a highly effective treatment for GPP flares in adults.
C. The following biologics: IL-17 inhibitors, IL-23 inhibitors, TNFa inhibitors are contraindicated for GPP treatment
D. Antifungal therapy can be used as first line treatment for GPP
E. Oral antibiotic monotherapy may be used as treatment of GPP

**Answers:**

A. There are globally approved guidelines for the treatment of GPP – Incorrect. Currently, there are no globally approved guidelines for the treatment of GPP and substantial variations in the management of GPP exist throughout the world.^5^
B. Spesolimab, an anti-IL-36 receptor antibody, is the most specific agent and a highly effective treatment for GPP flares in adults – Correct. New therapeutic developments have focused on modulating the aberrantly increased interleukin 36 signaling in GPP.^2^ The anti-IL-36 receptor antibody spesolimab is the most specific agent which inhibits the IL-36R and prevents the IL-36 inflammatory cascade.^5^ The literature highlights that spesolimab is a highly effective treatment of GPP flares in adults.^2^ Many other potential anti-IL-36 pathway therapies (including Imsidolimab) for GPP are still in development and testing.^5^
C. The following biologics: IL-17 inhibitors, IL-23 inhibitors, TNFa inhibitors are contraindicated for GPP treatment – Incorrect. IL-17 inhibitors, IL12/23 inhibitors, IL-23 inhibitors, and TNFa inhibitors are used with success despite not being approved globally.^1^^,^^4^^,^^5^ Only in Japan, IL-17 inhibitors including secukinumab, ixekizumab, and brodalumab are licensed for the treatment of GPP.^5^ Our patient was successfully treated with ustekinumab and remains in remission for the last 8 months without any flares (Fig 3).

**Fig 3:**
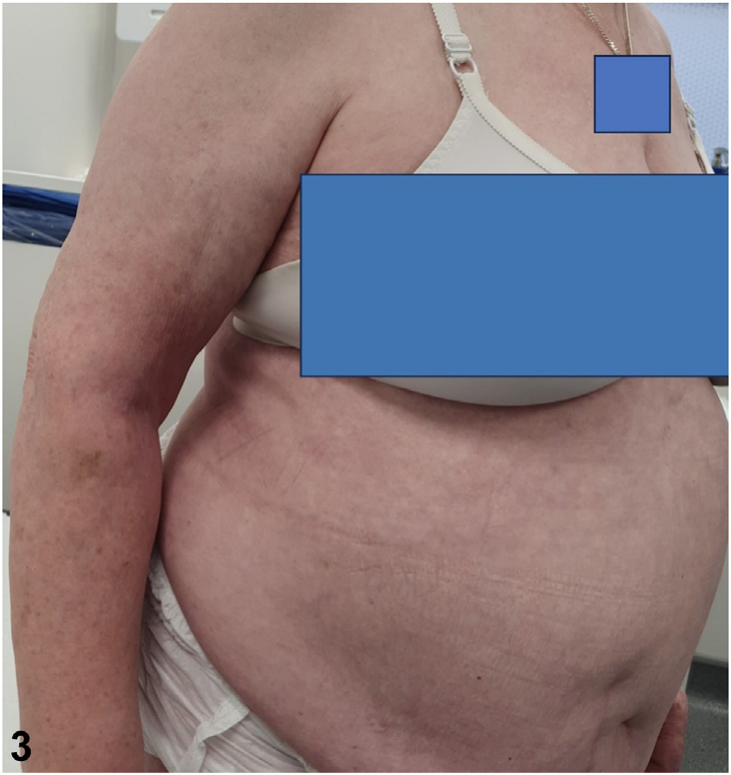
D. Antifungal therapy can be used as first line treatment for GPP – Incorrect. GPP is a rare chronic inflammatory skin condition.^5^ Antifungals are not used to treat GPP.
E. Oral antibiotic monotherapy can be used as treatment of GPP – Incorrect. GPP is a rare chronic inflammatory skin condition presenting with hundreds of grouped sterile pustules typically on an erythematous base.^5^ While infection can be a frequent complication, antibiotics should be considered only if superinfection is suspected, however they are not the main treatment.^1^

## Conflicts of interest

None declared.
